# PD13 Cost Effectiveness Of Imipenem-Cilastatin-Relebactam Compared With Colistina-Imipenem For Treating Confirmed Carbapenem Non-Susceptible Gram-Negative Bacterial Infections

**DOI:** 10.1017/S0266462324002812

**Published:** 2025-01-07

**Authors:** Martina Paoletti, Andrea Marcellusi, Joe Yang, Francesco Saverio Mennini

## Abstract

**Introduction:**

Antimicrobial resistance is an important public health problem with a strong epidemiological, economic, and social impact. The objective of this analysis was to compare the cost effectiveness of imipenem-cilastatin-relebactam (IMI/CIL/REL) therapy with colistin-imipenem for the treatment of hospitalized patients with Gram-negative bacterial infection caused by imipenem-resistant pathogens.

**Methods:**

The population comprised patients with hospital-acquired bacterial pneumonia or ventilator-associated bacterial pneumonia that was complicated with an intra-abdominal or urinary tract infection caused by carbapenem-resistant Gram-negative pathogens. The model started with a short-term decision tree describing possible treatment routes and outcomes for patients during hospitalization. After treatment, patients were classified as cured, not cured, or dead. Patients who had not responded to the initial treatment received another line of therapy. Successfully treated patients were entered into the long-term Markov model, which captured follow-up costs and health-related quality of life over their lifetimes.

**Results:**

The analysis was conducted on a hypothetical cohort of 1,000 patients and demonstrated that IMI/CIL/REL therapy was advantageous in terms of diagnosis and treatment in the short term as well as cost effectiveness. In fact, IMI/CIL/REL therapy was dominant, compared with colistin-imipenem, from the National Health System and the societal perspective, providing an average saving of EUR2,800.15 and EUR3,174.63, respectively, and gains of 4.76 years of life and 4.12 quality-adjusted life-years per patient.

**Conclusions:**

Thanks to its economic and societal value, IMI/CIL/REL therapy represents an investment in health that is lifesaving in critically ill patients and is a valuable public health tool in the fight against antimicrobial resistance.

